# Essential Oils and Extracts from Epazote (*Dysphania ambrosioides*): A Phytochemical Treasure with Multiple Applications

**DOI:** 10.3390/plants14131903

**Published:** 2025-06-20

**Authors:** Arsenio Heredia Severino, Juana Fernández-López, Fernando Borrás-Rocher, Manuel Viuda-Martos

**Affiliations:** 1 Facultad de Ciencias Agronómicas y Veterinarias, Universidad Autónoma de Santo Domingo-UASD, Santo Domingo 10105, Dominican Republic; bioarsenio@gmail.com; 2 Instituto Dominicano de Investigaciones Agropecuarias y Forestales-IDIAF, Santo Domingo 10205, Dominican Republic; 3 IPOA Research Group, Institute on Agri-Food and Agri-Environmental Research and Innovation (CIAGRO-UMH), Miguel Hernández University, 03312 Orihuela, Spain; j.fernandez@umh.es; 4 Statistics and Operative Research Department, Miguel Hernández University, 03202 Elche, Spain; f.borras@umh.es

**Keywords:** *Dysphania ambrosioides*, epazote, medicinal plants, bioactive compounds, pharmacological activity, phytotherapy, *Chenopodium ambrosioides*

## Abstract

*Dysphania ambrosioides*, commonly known as epazote, is a medicinal plant of great relevance in traditional Latin American medicine. Its cultural roots and pharmacological properties have made it an object of study for phytochemical research. An artificial intelligence (AI) tool was utilized to assist in reviewing scientific information regarding *D. ambrosioides*. An initial search was conducted in the Scopus database using the keywords epazote, *D. ambrosioides*, anti-helminthic, antioxidant, and antimicrobial, which yielded a total of 814 publications. To select the most relevant articles, this AI tool based on natural language processing (available online and free of charge) was applied, which analyzed the keywords that appeared in the titles and abstracts of the works and clustered them, leading to a reduction of 86.73% in the number of studies. *D. ambrosioides* stands out for its rich composition of bioactive compounds, which give the plant a wide range of therapeutic properties, including antiparasitic activity, through which it is effective against several parasites, such as helminths and protozoa, due to its schistosomicidal, nematocidal and antimalarial action. Additionally, it has shown antimicrobial, antioxidant, and anticancer properties as it contains compounds that help fight cell damage caused by free radicals. Epazote represents a rich source of compounds with a wide therapeutic range. However, much research is required to understand the mechanisms of action of these compounds and to evaluate their safety and efficacy in clinical trials.

## 1. Introduction

The *Dysphania ambrosioides* (L.) Mosyakin & Clemants, otherwise known as Jesuit’s tea, Mexican tea, payqu (paico), epazote, mastruz, or herba sanctæ Mariæ (Arabic: M’khinza, French: anserine vermifuge) [1], is a wild species from tropical America, naturalized in North America, Africa, Europe, Australia, and Asian countries [2]. It is an herbaceous plant of the genus *Dysphania*, belonging to the family *Amaranthaceae* [3].

**Figure 1:**
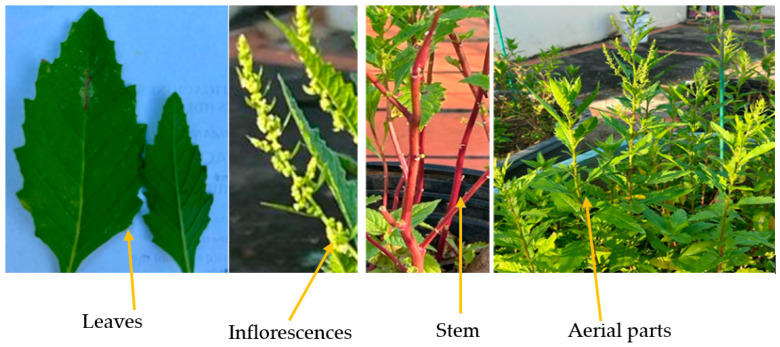
Different parts of the plant *Dysphania ambrosioides*.

This plant (Figure 1) reaches up to 1 m in height, and it shows annual or perennial growth. It features stems that may be single or branched, and its leaves grow in an alternating pattern along the stem [4]. These leaves ranged from narrowly elliptic to elliptic and are green, oblong–lanceolate with the smallest leaves located at the top of the plant, directly attached to the stem (sessile), while the largest leaves are found at the base of the plant and have short petioles (leaf stalks). The flowers are small and green with dense terminal panicles of glomeruli, with five sepals. The plant is known for its strong and distinctive aroma. Its fruits and seeds are black and horizontal and are enclosed in a persistent calyx that is usually less than 0.8 mm long [5,6].

**Figure 2:**
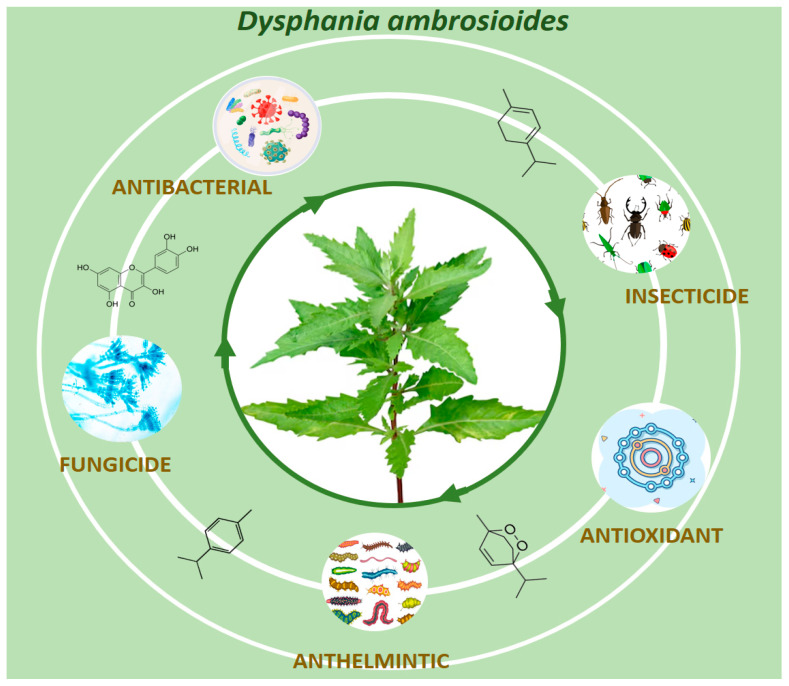
Biological activities of *Dysphania ambrosioides*.

The World Health Organization (WHO) reported that *D. ambrosioides* is among the most widely used medicinal plants around the world [1]. In Latin America, this plant is used in folk medicine because it has anti-helminthic, vermifuge, antispasmodic, and antipyretic activity. Additionally, it is used in the treatment of dental and digestive ailments, as well as in skin disorders, including dermal wounds and eczema [7,8,9]. In addition, the decoction, infusion, and maceration of leaves, stems, and branches were used in the treatment of respiratory disorders, cough, expectorant, and musculoskeletal injury [10]. In the Dominican Republic, the epazote is a shrubby plant that grows in the yard sporadically and has been used for the treatment of intestinal worms for centuries. In addition, this plant and its extracts have demonstrated several interesting biological activities such as antifungal, antioxidant, antibacterial, insecticide, and anti-helminthic effects (Figure 2). Other biological activities include anti-inflammatory activity, inhibiting cell proliferation, suppressing tumor growth, preventing cancer development, and inducing programmed cell death [11,12].

The use of plants for the treatment of diseases is nearly as ancient as the human species itself. They produce a diverse array of secondary metabolites, including vitamins, tannins, polyphenols, alkaloids, terpenoids, and flavonoids, among others [13], which exhibit useful biological activities such as antimicrobial, antiparasitic, and antioxidant activities. However, it is essential to conduct a phytochemical characterization to identify the biological activity of each active compound, as this will elucidate their respective advantages and disadvantages. On the other hand, there is an increasing interest in developing natural and environmentally friendly derived alternatives for the control of insect pests, bacteria, parasites, and fungi [14]. So, plant derivatives represent a potential alternative in this context, being currently at the forefront of novel and promising drug development [15].

Scientists often face the challenge of conducting literature reviews on topics that encompass hundreds or even thousands of articles. In these cases, the time and effort required to conduct it correctly can be overwhelming. Additionally, the process can often be influenced by the authors’ backgrounds and knowledge, which can introduce selection bias and result in varying versions of the state of the art depending on the individual researchers. Trying to address this issue more efficiently and objectively, this study applies an easily accessible technological tool (available for free) based on artificial intelligence (AI) and natural language processing (NLP), which has been successfully applied in several literature review processes on topics related to food science [16] and biological activity [17]. This tool enables users to compare and analyze the state of the art in a more structured and objective manner, leading to a more reliable review process.

This review aims to summarize the current knowledge on the use of the *Dysphania ambrosioides* plant, highlighting its chemical composition, antibacterial, antifungal, antioxidant, antiparasitic, and insecticidal activity, as well as its potential applications. For the selection of the relevant information to be deeply studied and analyzed, an AI-based program was used.

## 2. Methodology

### 2.1. Bibliographic Search Conditions

This review was based on a bibliographic search using the SCOPUS and Web of Science databases that offers a comprehensive overview of global interdisciplinary scientific information, covering the areas of science, technology, and medicine (among others) in which the specific topic under study is framed. From this search, information about the titles of the papers, year of publication, abstract, and keywords was obtained. For this basic search, the following search conditions were selected: “epazote”, “Dysphania”, “ambrosioides”, and “Mexican Tea as the search topic; “review” and “article” as the document type; and “from 2015 to 2024” as the limit period for the search. No other filter or search restriction was applied. From this basic search, a file with 814 documents was obtained. From these documents, the search provided information on the titles of the articles, their year of publication, their abstracts, and keywords. This huge amount of information would require a highly time-consuming process to select a reasonable number of relevant papers to review in depth and obtain the required information about the aim of this review. To avoid that, it was decided to apply a new AI tool based on the NLP [16,17].

### 2.2. Selection Process of Relevant Papers Assisted by the AI Tool

This new AI tool has been specifically developed to assist in this process of selecting relevant information. The Jupiter notebook on Google Colab in this tool is freely available (Table S1) and has already been successfully applied with the same objective for a biology-related topic [17] and a food-related topic [16]. This new tool automatically assesses and categorizes all the documents by examining the content of their titles and abstracts, thus enabling the clustering of such a huge amount of bibliographic information [18]. Briefly, the operation of such an algorithm includes the steps described below.

#### 2.2.1. Preprocessing of Search Data

**Figure 3:**
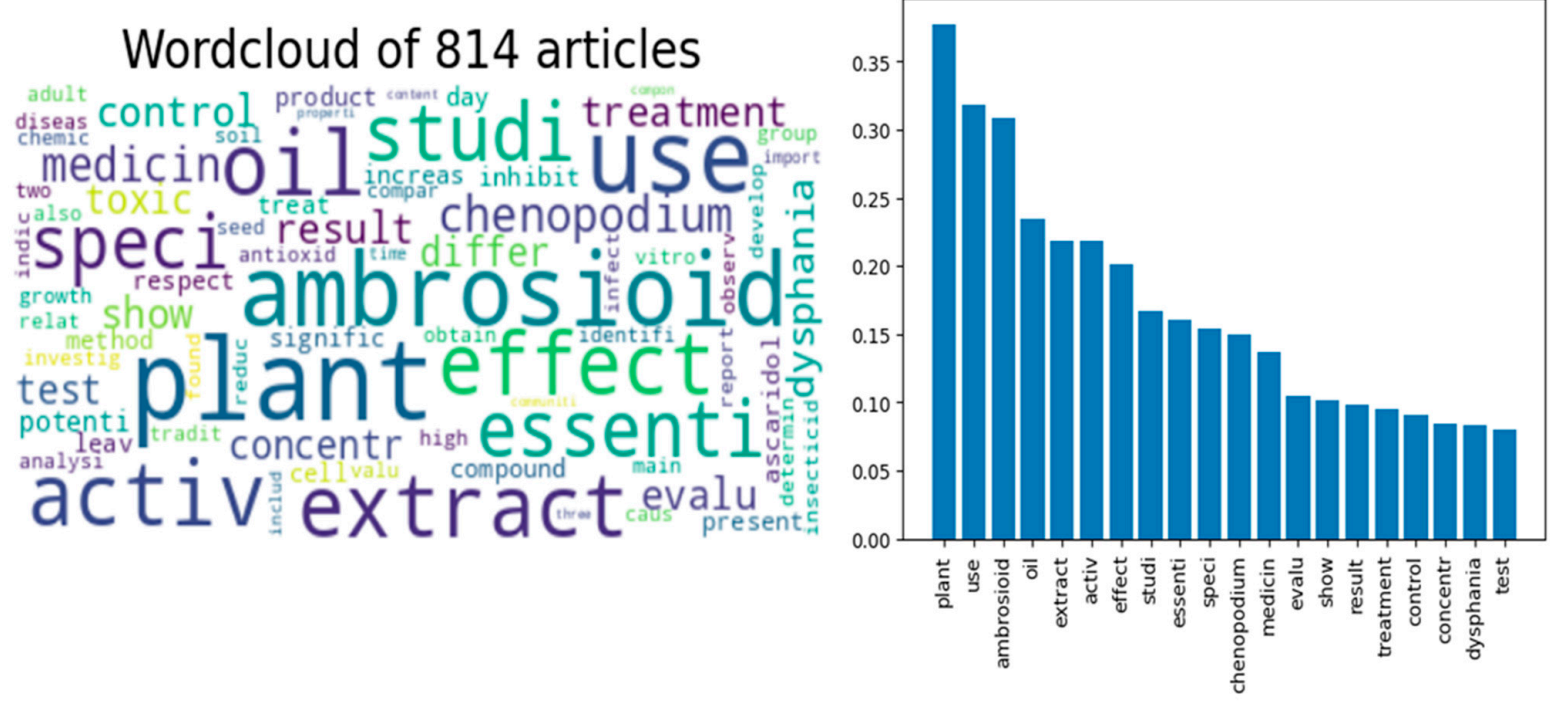
Word cloud of the 75 most common words from the 814 papers about *Dysphania ambrosioides* and the corresponding frequency bar chart.

The data from the Scopus and Web of Science files required preprocessing before analysis to convert unstructured text data into normalized and structured data using the Python (version 3.12 for windows) Natural Language Toolkit library, which involved 3 steps: noise removal, normalization, and tokenization. After that, exploratory analysis tools were used to aggregate and visualize the text data associated with the 814 contributions obtained from the initial search (which lasted a total of 200 s), providing a bar plot and word cloud as a visual representation of the most frequently occurring words (75 words) in the abstracts used (Figure 3), which was very useful for selecting the most relevant articles. These graphical representations allowed for an understanding of the data to verify the preprocessing stage, ensuring that the analysis was on the right track, or alternatively, determining whether further preprocessing would be necessary before training the model.

#### 2.2.2. Text Clustering and Cluster Analyses

For text clustering, the tool uses the k-means algorithm, a simple and popular unsupervised clustering algorithm [19], in order to find groups of similar abstracts in the initial search file. This algorithm measures the distance between points (in this case, the abstracts of articles) and groups those that are close together, signifying their similarity. An effective cluster is characterized by having smaller distances between its points compared to the distances between points in separate clusters. Determining the optimal number of clusters is essential for organizing the information. To achieve this, two primary methods were employed: the elbow method and the average silhouette method [18,19,20]. In addition, this tool offers insightful details about the resulting clusters, including their relationships, proximity, temporal distribution, and other relevant aspects, the analysis of which allowed us to evaluate the resulting categorization or clustering and select the clusters that were considered to best represent the initial objective of the proposed review. This process can be repeated as many times as necessary to obtain a reasonable number of articles that will then be subjected to the traditional review process.

## 3. Selection of the Relevant Papers Assisted by the AI Tool

The optimal number of clusters obtained in the first analysis was 19 (numbered from 0 to 18). In this case, for the 19 clusters obtained, the information provided was the number of papers associated with each cluster (Figure S1); the corresponding word clouds (Figure S2) and figure bars (Figure S3) of the top words of each cluster; the results of the principal component analysis showing the relationship among the 19 clusters (Figure S4); and the distribution of the 19 clusters over the time (Figure S5).

From the analysis of all this information, clusters 0 (17 papers), 1 (79 papers), 3 (40 papers), 6 (60 papers), 7 (42 papers), 10 (68 papers), and 15 (96 papers) were selected as the most relevant for the study, mainly based on the analysis of the top words (Figures S2 and S3). In all of them, the words “extract”, “plant”, “activity”, “nematode”, “infection”, “insecticide”, “oil”, “essential”, or “compound” appeared as top words. By contrast, clusters in which words such as “seed”, “germination”, “infusion”, “volatiles”, “medicine”, “family”, “food”, “soil”, “tolerance”, “nanoparticles”, “metal”, “toxicity”, “leishmaniosis” or “temperature” appeared as top words were not selected because they were not related to the aim of the present work. So, a total of 402 papers were included in the selected clusters, representing a reduction of 51% compared to the number of papers resulting from the initial search (814 papers). Nevertheless, it was still an unmanageable number of articles, so the initial process was repeated on these 402 papers (Table S1; second round). In this case, 19 subclusters were again obtained (also providing the same information as in the first round) (Table S1). In this second round, and from the analysis of these 19 subclusters, 3 of them were selected as relevant: subcluster 4 (71 papers included), subcluster 6 (76 papers included), and subcluster 14 (36 papers included). So, in this second round, a total of 179 documents were selected as relevant, indicating a total reduction of 78% compared to the initial search (814 papers). The word cloud of the top words included in each of these three subclusters is shown in Figure 2. It can be observed that the top words in these subclusters are “antimicrobial”, “antibacterial”, “antioxidant”, “insecticide”, “nematode”, “activity”, “control”, “extract”, “essential”, “oil”, “plant”, and “*ambrosioides*”, all of them highly related with the aim of this review. The number of papers included in these three subclusters (179) was considered feasible and allowed us to proceed with a traditional review, consisting of reading each paper in depth and extracting the most relevant results.

In the process of downloading documents, 32 documents were eliminated because they were repeated or they were not available as a publication in line and it was not able to obtain the full text. Therefore, the final number of papers that were downloaded and analyzed was 147. Of these, 23 papers were still discarded because they did not provide useful information for this study, so the final papers number included in this review was 124.

## 4. Traditional Review of the Selected Papers

### 4.1. Chemical Composition of Dysphania ambrosioides

The essential oils obtained from the different parts (leaves, stems, roots, and flowers) of *D. ambrosioides*, a species extensively used in traditional medicine in Costa Rica, were extensively analyzed, using several methodologies, including gas chromatography–mass spectrometry, gas chromatography–mass spectrometry tandem, and gas chromatography FID. The main components of essential oil obtained from this plant can be classified as monoterpenes and sesquiterpenes (as hydrocarbons, alcohols, ketones, etc., which may be acyclic, monocyclic, bicyclics, or tricyclics) [21]. In the same way, it is also possible to obtain ethanolic or methanolic extracts with a high content of bioactive compounds, mainly phenolic acids and flavonoids [6]. In the scientific literature, there were several studies where the chemical composition of *D. ambrosioides* essential oil had been determined, as shown in Table 1 [22,23,24,25,26,27,28,29,30,31,32,33,34,35,36,37,38,39,40,41,42,43,44,45,46]. Nevertheless, it should be noted that the composition and concentration of compounds depend on several factors, such as the part of the plant used, the methodology used to obtain the extracts, the environmental conditions, and the harvest period, as well as genetic factors [47]. In this sense, Fdil et al. [48] identified *p*-cymene (41.7%), α-terpinene (34.8%), and ascaridole (10.8%) as major constituents of the essential oil obtained from leaves of *D. ambrosioides* collected in Morocco. Fatokun et al. [5] reported that the essential oil extracted from fresh leaves of *D. ambrosioides* by hydrodistillation had a total of twenty compounds with γ-terpinene (48.68%), *o*-cymene (21.71%), *trans*-β-terpinyl butanoate (17.15%), and ascaridole (5.67%), which were the major compounds identified. Kandsi et al. [32] determined the composition of essential oils obtained by hydrodistillation of the aerial components of *D. ambrosioides* growing in Eastern Morocco. These authors reported the most abundant compounds in essential oil are (+)-4-carene (50.5%), α-cyclogeraniol acetate (22.64%), and (1R,2R,3R,5S)-(−)-isopinocampheol (18.87%), respectively.

Hsu et al. [23] analyzed the oil obtained from the leaves of *D. ambrosioides* cultivated in Taiwan, and it was found to consist of α-terpinene (30.5%), *p*-cymene (17.3%), carvacrol (16.2%), and ascaridole (15.1%). In a similar study, Maldaner et al. [22] carried out a study to analyze the chemical composition of essential oil obtained by hydrodistillation of young leaves of *D. ambrosides* from the Amazon region in Brazil. These authors reported that the main components were oxygenated monoterpenes such as ascaridole, ascaridole glycol, linalool acetate, and dihydrocitronellol acetate, with values of 5.75, 10.58, 11.26, and 19.53%. Similarly, Ez-Zriouli et al. [29] reported that the essential oils obtained from aerial parts *of D. ambrosioides* cultivated in the Region of Safi in Morocco were very rich in monoterpene peroxides and monoterpenes, including α-terpinene (53.4%), ascaridole (17.7%), and *p*-cymene (12.1%). In the same way, Azghar et al. [30] studied the composition of essential oils obtained by hydrodistillation of the aerial part of *D. ambrosioides* collected in the Region of Eastern Morocco, and they reported that *p*-cymene (31.72%), 4-carene (27.34%), and α-cyclogeraniol acetate (16.90%) were the main components.

As mentioned above, the ethanolic or methanolic extracts obtained from several parts of the plant have also been analyzed to determine the polyphenolic profile. Therefore, the analysis by HPLC-DAD revealed the presence of rutin in the crude extract (12.5 mg/g), ethyl acetate (16.5 mg/g), and *n*-butanol (8.85 mg/g), whereas quercetin and chrysin were quantified in chloroform fraction (1.95 and 1.04 mg/g), respectively [49]. Kandsi et al. [1] carried out research to analyze the polyphenolic profile of *D. ambrosioides* flower hydroethanolic extract. These authors reported that the hydroethanolic extract contains mainly syringic acid, quercetin, hesperetin, and luteolin. In a similar study, Li et al. [50] analyzed the polyphenolic profile of ethanolic extract obtained from the aerial part of *D. ambrosioides* using an ultrasonic-assisted extractor. They found that the main components were kaempferitin, kaempferol-3-*O*-apigenin-7-*O*-rhamnoside, and kaempferol-3-*O*-acetylapigenin-7-*O*-rhamnoside, with a concentration of 33.5, 60.77 and 5.73%, respectively. More recently, Figueroa-Merma et al. [51] analyzed the polyphenolic profile of an extract obtained from aerial parts of *D. ambrosioides* plants grown in Lima. They found that the main components were Kaempferol rhamnosyl-dipentoside and Kaempferol dirhamnoside-hexoside, with values of 160.00 and 155.59 mg/100 g dry weight. Another group of bioactive compounds present in the extracts obtained from aerial parts of *D. ambrosioides* is alkaloids, which are found in high concentrations in this plant, as reported by various authors [49,52]. In this sense, Shah et al. [53] stated that *D. ambrosioides* is rich in 1-Piperoylpiperidine. Another study carried out by Kandsi et al. [52] revealed that the hydroethanolic extracts obtained from the flowers of *D. ambrosioides* are a rich source of alkaloids, such as trisphaeridine, galanthamine, crinine, demethylmaritidine, anhydrolycorine, nor-galanthamine, N-formylnorgalanthamine, peramine, and ergovaline. Figueroa-Merma [51] reported that α-, β-, γ- and δ-tocopherols were found in amounts of 28.78, 7.19, 7.96, and 3.27 µg/g in extracts obtained from leaves of D. ambrosioides cultivated in Peru. Previously, Shah and Kanh [53] mentioned that the main phytosterols found in methanolic extracts of *D. ambrosioides* were stigmasterol and β-sitosterol. Coumarins were also present in the extracts obtained from the aerial part of D. ambrosioides; thus, Ghareeb et al. [54] reported the presence of 1,2-benzopyrone in the extracts obtained from the leaves of D. ambrosioides, while Shah and Khan [53] found that scopoletin is present in methanolic extracts obtained from leaves.

### 4.2. Antimicrobial Properties of Dysphania ambrosioides

The extracts and essential oils obtained from several parts of *Dysphania ambrosioides* have been shown to exhibit antimicrobial activities against various microorganisms, including bacteria, yeasts, and fungi, as shown in Table 2 [41,49,55,56,57,58,59,60]. These activities make them potential natural preservatives for food and pharmaceutical products, as well as agents for the treatment of microbial infections [29]. In reference to essential oils, De Andrade Santiago et al. [61] assessed the antibacterial properties of essential oil extracted from leaves of *D. ambrosioides*, whose principal components were α-terpinene and *r*-cymene, against several bacteria strains, including *Staphylococcus aureus*, *Listeria monocytogenes*, *Escherichia coli* and *Salmonella cholerasuis*. These authors reported minimum inhibitory concentration (MIC) values of 62.5, 250, 125, and 125 µg/mL, respectively. Almeida Bezerra et al. [28] reported that the essential oils that had a high content of α-terpinene and ascaridole, obtained from leaves of *D. ambrosioides* cultivated in Brazil, demonstrated significant MIC values against *S. aureus* (256 µg/mL), moderate values against *Pseudomonas aeruginosa* (512 µg/mL) and low values against *E. coli* (1024 µg/mL). Kandsi et al. [32] carried out a study to analyze the antibacterial activity against *E. coli*, *S. aureus,* and *Enterococcus faecalis* of essential oil obtained from the stem and flowers of *D. ambrosioides* cultivated in Morocco. These authors reported that the MIC values of essential oil obtained from the stem were 18, 18, and >110 µg/mL for *E. coli*, *S. aureus*, and *E. faecalis*, respectively, while for the essential oil obtained for the flowers, the MIC values were 6,12, and 105 µg/mL for *E. coli*, *S. aureus*, and *E. faecalis*, respectively. This antibacterial activity could be due to the high content of 4-carene and α-cyclogeraniol acetate present in essential oil. In a similar study, Azghar et al. [30] studied the effect of essential oil (rich in *p*-cymene and 4-carene) obtained from the aerial part of *D. ambrosioides* on multidrug-resistant *E. coli*, *Acinetobacter baumannii*, *P. aeruginosa*, and methicillin-resistant *S. aureus*, reporting MIC values of 150,120, 140 and 230 µg/mL, respectively.

The extracts (ethanolic, methanolic, aqueous, and n-hexane) obtained from the different parts of the *D. ambrosioides* plant also have demonstrated antibacterial activity against several Gram+ and Gram− strains. In this context, Knauth et al. [59] reported that the methanolic extract from the fruit of *D. ambrosioides* cultivated in Mexico, rich in flavonoids, inhibited the bacterium *Enterococcus faecalis*, *E. coli*, and *Salmonella typhimurium*, with MIC values of 4375, 1094, and 137 µg/mL, respectively. More recently, Ouadja et al. [62] analyzed the antibacterial activity of the ethanolic extract from the leaves of *D. ambrosioides* cultivated in Togo, with a high content of phytol. They reported MIC values of 500 mg/mL against *S. aureus*, *P. aeruginosa*, and *Citrobacter freundii*. Similarly, Bano et al. [63] reported that the n-hexane extracts (100 µg/disc) obtained from *D. ambrosioides* seeds cultivated in Pakistan had high antibacterial activity against *Klebsiella pneumonia*, *Micrococcus luteus*, and *S. aureus*, with inhibition halos of 14, 13, and 9 mm, respectively. This activity probably could be due to the high content of quercetin and kaempferol found in the extracts. Martínez-Alva et al. [58] reported the use of leaf extracts from *D. ambrosioides* cultivated in Mexico City, rich in alkaloids, which showed antibacterial activity against *Clostridioides difficile*, with a MIC value of 3900 µg/mL. The antibacterial activity of *Dysphania* extract and essential oil makes it a promising natural alternative to synthetic bactericides. The oil could be used as a disinfectant in healthcare settings, reducing the risk of hospital-acquired infections [55]. It could also be used in food preservation, extending the shelf life of perishable products and reducing the risk of foodborne illnesses [59]. Additionally, the extracts could be used in pharmaceutical applications, such as in the development of novel antibacterial agents. The antibacterial activity of *Dysphania* is attributed to the synergistic effects among several compounds. Terpenes, such as α-terpinene and *o*-cymene, are known to disrupt the bacterial cell membrane, leading to leakage of cellular contents and ultimately, cell death [64]. In addition, as mentioned by Musa et al. [65] and Singh and Pandey [2], the bioactive compounds present in *D. ambrosioides* are mainly hydrophobic, which allows them to enter the bacterial cell membrane and mitochondria, disrupt the cellular structure, and produce the death of bacteria. In addition to antibacterial activity, extracts or essential oils obtained from *D. ambrosioides* have also been shown to possess significant antifungal activity. Therefore, in the scientific literature, it is possible to find several studies where the antifungal activity of *D. ambrosioides* extracts or essential oil has been determined. Stappen et al. [34] assessed the antifungal activity of the essential oil obtained from leaves and inflorescence of *D. ambrosioides* cultivated in western Himalaya against *Colletotrichum gloeosporioides*, *Colletotrichum acutatum*, and *Colletotrichum fragariae.* They found that the inhibition zones of fungal growth were between 6.5 and 8.0 mm when concentrations of 80 µg/spot were used, and inhibition zones of 11.0 to 14.5 mm were observed for concentrations of 160 µg/spot. Mokni et al. [43] evaluated the antifungal activity of the essential oil obtained from fresh leaves of *D. ambrosioides* collected in northwestern Tunisia. These authors informed that this essential oil showed antifungal activity against the pathogenic strain *Candida albicans* yeast, with MIC values equal to 39 µg/mL. In a similar study, Almeida Bezerra et al. [28] evaluated the cellular viability of different strains of *C. albicans* and *Candida tropicalis* when exposed to essential oil obtained from the leaves of *D. ambrosioides* cultivated in Brazil. It is possible to notice that, for *C. albicans* LM77, the essential oil had an IC_50_ value of 19.3 µg/mL, while for *C. albicans* INCQS 40006, the IC_50_ value was 25.2 µg/mL. Regarding the cellular viability of *C. tropicalis* LM 23, the essential oil showed antifungal activity, with an IC_50_ of 101.9 µg/mL and an IC_50_ of 15.8 µg/mL for the *C. tropicalis* INCQS 40042 strains. Zefzoufi et al. [21] found that the essential oil obtained from leaves at µg/mL inhibited the growth of *Pseudomonas syringae* pv. *syringae*, *P. syringae* pv. *tabaci*, and *Erwinya amylovora.* More recently, Hsu et al. [23] carried out a study to analyze the antifungal activity against wood decay fungi of essential oils obtained from fresh leaves of *D. ambrosioides* cultivated in Taiwan. They reported a complete inhibition of the fungal *Phaeolus schweinitzii* and *Lenzites sulphureus*, with a concentration of essential oil of 50 µg/mL, while for *Phaneochaete chrysosporium*, the concentration required was 100 µg/mL. Finally, for *Trametes versicolor*, 200 µg/mL of essential oil was necessary for inhibition. In reference to the MIC values, these authors reported values of 0.10, 0.10, 0.10, and 0.05 mg/mL for *T. versicolor*, *P. schweinitzii*, *P. chrysosporium*, and *L. sulphureus*, respectively.

The antifungal properties of extracts obtained from *D. ambrosioides* have also been determined. In this sense, Bano et al. [63] reported that chloroform and acetone–methanol *D. ambrosioides* seed extracts had great activity against *Fusarium solani* and *Aspergillus fumigatus*, with inhibition zones of 17 and 12 mm, respectively. These authors reported MIC values of 100 µg/disc against *Aspergillus niger*, *A. fumigatus, Aspergillus flavus, Fusarium solani*, and *Mucor* spp. Similarly, Gishen et al. [57] conducted a study to analyze the antifungal activity against *C. albica* of ethanolic extracts obtained from fresh leaves of *D. ambrosioides* cultivated in Ethiopia. They found that the analyzed extract showed a minimum inhibition zone concentration of 1 g/mL. The high antifungal activity of *D. ambrosioides* might be due to the essential oil and extracts having a high amount of ascaridole in their composition. This is a bicyclic monoterpene with a rather unusual bridging peroxide functional group and has long been the only known natural peroxide [34]. Moreover, besides the main ingredients, minority components can also play an important role in the antifungal activity of extracts. Multiple botanical chemicals and essential oils present in plants may exert synergistic or antagonistic effects [63]. Phenolic compounds might be responsible for antifungal activity. From our perspective, and based on the results reported in the analyzed papers, *D. ambrosioides* essential oil is not the most effective plant-derived essential oil for controlling bacteria and fungi. However, it is a viable alternative for extending the shelf life of various foods, due to its ability to reduce microorganism growth. It should be noted that its activity is reduced when applied to several matrices. On the other hand, given its properties, we believe its use would be valuable in barrier technology for food preservation.

### 4.3. Antioxidant Properties of Dysphania ambrosioides

*Dysphania ambrosioides* extracts and essential oils have been found to possess antioxidant and anti-inflammatory properties, which can help protect against oxidative stress and inflammation-related diseases. These activities make them potential natural remedies for the prevention and treatment of chronic diseases such as cancer, cardiovascular disease, and neurodegenerative disorders [7,66].

There exists a marked interest in natural antioxidants, particularly in compounds such as flavonoids and other polyphenols, including tannins, abundantly present in plants like *D. ambrosioides*. In the scientific literature, it was possible to find several studies where the antioxidant activity of *D. ambrosioides* extracts or essential oil obtained from different parts of the plant, including the aerial part, flowers, seeds, stems, and leaves, had been determined, as shown in Table 3 [60,63,66,67,68,69].

De Andrade Santiago et al. [61] assessed the antioxidant capacity of essential oil extracted from leaves of *D. ambrosioides*, whose principal components were α-terpinene and *r*-cymene, using two different methodologies such as the β-carotene–linoleic acid and DPPH assays. These authors reported an IC_50_ value in the β-carotene–linoleic acid test of 455.7 µg/mL, while for the DPPH assay, 500 µg/mL resulted in the inhibition of radical DPPH by 15.79%. Villalobos-Delgado et al. [70] studied the antioxidant activity of ethanolic extract (1 g/20 mL) obtained from leaves of *D. ambrosioides* cultivated in Mexico and reported the inhibition of the radical measured with the DPPH assay by 16.65%. This antioxidant activity could be due to the high content of quercetin and kaempferol *o*-rhamnosyl-pentoside present in the extract. Ogunleye et al. [11] conducted a study to analyze the antioxidant properties of extracts obtained from the aerial parts of *D. ambrosioides* cultivated in Nigeria, which showed a high concentration of 16-methyl-heptadecane-1,2-diol and phytol. The antioxidant assays revealed that *D. ambrosioides* leaf extracts possess antioxidant properties, with hexane fraction exhibiting the highest scavenging activities for DPPH, with an IC_50_ value of 0.02 mg/mL, while in the FRAP assay, the value obtained was 730.92 mg ascorbic acid equivalent/g. Ouadja et al. [62] determined the antioxidant capacity of hydroethanolic extract, with a high content of phytol obtained from the leaves of *D. ambrosioides* cultivated in Togo using three different methodologies, namely FRAP and ABTS assays. They reported antioxidant activity values of 32.48 and 45.33 µg ascorbic acid equivalent/mg for samples subjected to the FRAP and ABTS assays, respectively. In this sense, Pandiangan et al. [71] analyzed the antioxidant activity (DPPH assay) of water and acetone extracts obtained from the leaves of *D. ambrosioides* cultivated in Indonesia. They reported that the IC_50_ value of the acetone extract of *D. ambrosioides* was 9.7 µg/mL, while the IC_50_ value of the water extract was 1.32 µg/mL. Tchani et al. [72] analyzed the antioxidant activity of ethanolic and aqueous extracts, which showed a high concentration of flavonoids, obtained from the leaves and seeds of *D. ambrosioides* by maceration and infusion. These authors revealed that infusion yielded IC_50_ values of 25.541 and 48.269 µg/mL in aqueous and ethanolic media, respectively, whereas maceration resulted in IC_50_ values of 29.18 and 50.99 µg/mL, respectively. In a similar study, Kandsi et al. [32] analyzed the antioxidant properties of essential oils obtained from *D. ambrosioides* leaves cultivated in Morocco at concentrations ranging between 25 and 400 µg/mL using the DPPH and β-carotene assays. They reported IC_50_ values of 210.24 and 220.50 µg/mL for DPPH and β-carotene assays, respectively. This antioxidant activity could be due to the content of 4-carene and α-cyclogeraniol acetate. In this way, Bano et al. [63] reported that the antioxidant capacity of methanolic extracts, which showed a high content of quercetin and kaempferol, obtained from *D. ambrosioides* seeds cultivated in Pakistan and measured with ABTS and FRAP assays, was 110.6 and 94.3 µg ascorbic acid equivalents/mg extract, respectively, while the *n*-hexane extract revealed the lowest antioxidant potential, with values of 11.2 and 13.7 ascorbic acid equivalents/mg extract for ABTS and FRAP assays, respectively. Ez-Zriouli et al. [29] investigated the antioxidant activity of essential oil rich in ascaridole and α-terpinene, which was obtained from the aerial part *D. ambrosioides* collected in Morocco, and they reported values of 30.82 mg Trolox equivalent per g of essential oil. More recently, Drioua et al. [66] assessed the antioxidant activity of ethyl acetate fraction from the aerial components of *D. ambrosioides* cultivated in Morocco, which showed a high content of flavonoids and phenolic acids, employing the DPPH assay. They reported that these extracts yielded an IC_50_ value of 0.54 mg/mL.

### 4.4. Insecticidal and Repellent Activities of Dysphania ambrosioides

The extracts and essential oils obtained from several plants have been used for insect control for centuries around the world and are considered safe due to their relatively short shelf life and low toxicity to humans and animals [73]. In this way, the extracts and essential oils of *D. ambrosioides* have been shown to possess insecticidal and repellent activities against mosquitoes and other insects, making them potential natural insecticides and repellents for the control of vector-borne diseases [34], as essential oils negatively affect the feeding, growth, reproduction, and oviposition of harmful insects [73]. In the scientific literature, there were several studies [34,37,38,73,74,75,76,77,78,79,80] where the insecticidal and repellent activities of *D. ambrosioides* extracts or essential oil had been determined (Table 4).

Vite-Vallejo et al. [78] evaluated insecticidal activity against *Bemisia tabaci* using ethanolic extracts obtained from *D. ambrosioides*, cultivated in Mexico. They reported that the extracts of *D. ambrosioides* at concentrations of 1, 2, 3, 4, 5, and 6% killed 3.5, 10, 26, 70, 88, and 93% of *B. tabaci*, respectively. In a similar study, Stappen et al. [34] analyzed the insecticidal activity of essential oils rich in ascaridole and p-cymene, obtained from the leaves of *D. ambrosioides* collected from India, against *Aedes aegypti*. These authors mentioned that *D. ambrosioides* essential oil had a mortality of 100%, with a concentration of 125 mg/L, while the mortality at a concentration of 62.5 mg/L was 80%. Langsi et al. [81] reported that the essential oil obtained from leaves of *D. ambrosioides*, which showed a high content in 4-carene and p-cymene, caused at least 80% *Sitophilus zeamais* mortality within 14 days of storage with a dose of 200 µL/kg. In addition, 8 µL of essential oil was repellent to the weevils. Velez et al. [77] reported that the extract obtained from the leaf powder of *D. ambrosioides* grown in Brazil showed insecticidal activity against *Dactylopius opuntiae*. They reported that aqueous *D. ambrosioides* extract at 10% had a corrected mortality of 24.41%, while the corrected mortality of hydroethanolic *D. ambrosioides* extract at 5% was 17.49%. Almadiy [38] conducted a study to analyze insecticidal activity against the larvae and adults of *Culex quinquefasciatus* using essential oils obtained by hydrodistillation from aerial parts of *D. ambrosioides* grown in Saudi Arabia, which had a high concentration of *(Z)*-ascaridole. These authors reported that the larval mortality varied between 16.30 and 30.34% when the lower concentration (3.125 µL/L) was used, while for the higher tested concentrations (50 µL/L), the mortality increased to 80.11–100.00% after 24 h of treatment. Finally, they found that all treatments for larval and adult mortality were time- and dose-dependent. More recently, Laghzaoui et al. [74] reported that the essential oils obtained from leaves of *D. ambrosioides* cultivated in Morocco were toxic to the adult males and crawlers of *Dactylopius opuntiae*. The authors reported that this essential oil, which had a high content of isoascaridole and carvacrol, showed an LC_50_ and 90% lethal concentration (LC_90_) of 0.004 and 0.009 µL/cm^2^, respectively, against adult males of *D. opuntiae* using contact bioassay. On the other hand, the values obtained for LC_50_ and LC_90_ of essential oil against crawlers of *D. opuntiae* using contact bioassay were 0.003 and 0.018 µL/cm^2^, respectively. In a more recent study, Yikinç and Tunaz [73] analyzed the insecticidal activity of essential oils obtained from leaves of *D. ambrosioides* against *Periplaneta americana*. These authors found that higher mortality rates of *P. americana* adults occurred at a concentration of 5 µL/L of the essential oils derived from *D. ambrosioides*, while with a concentration of 2.5 µL/L of *D. ambroisoides* essential oil, after 24 h, 100% mortality was achieved for *P. americana* adults. Kasrati et al. [33] conducted a study to analyze the insecticidal activity of essential oils obtained from the leaves and inflorescences of *D. ambrosioides* against *Tribolium confusum* adults. These authors reported that these essential oils had an LD_50_ of 4.30 and 4.46 µL/L air and LD_90_ of 6.51 and 9.62 µL/L air for toxicity by fumigation. This activity could be due to the high content of δ-3-carene and *p*-cymene found in this essential oil.

### 4.5. Antiparasitic Activities of Dysphania ambrosioides

Annual yield losses in vegetables caused by phytonematodes in the world are estimated at 11% [82]. The use of *D. ambrosioides* extracts in the control of root-knot nematodes is even more important in family farming since horticulturists have low purchasing power and little access to more advanced technologies. Extracts and/or essential oils represent a technically viable and low-cost option compared to the main traditional control techniques [82]. The extracts and essential oils of *D. ambrosioides* yield promising results probably due to the complex mixtures of compounds from plant secondary metabolism that act as antimicrobial, antiviral, and antifeedant agents to protect plants [24].

Essential oils exhibit broad activity against parasitic microorganisms since they negatively affect the feeding, growth, reproduction, and oviposition of harmful parasites [73]. Based on the induction of different mitochondrial targets, many authors have attributed the antiparasitic activity to the major chemical components, namely carvacrol, caryophyllene oxide, and ascaridole [83,84]. The scientific literature presents several studies [42,45,82,85,86,87,88,89] where the antiparasitic activities of *D. ambrosioides* extracts or essential oil have been studied (Table 5).

In a study carried out by Guimarães et al. [82], antiparasitic activity against *Meloidogyne javanica* and *Abelmoschus esculentus* was analyzed using the extracts obtained from the leaves of *D. ambrosioides* collected in Pakistan. The plant extract of *D. ambrosioides* yielded a lower reproduction factor (FR = 11.80) of the nematode in the roots of the *Abelmoschus esculentus*. In addition, *M.* *javanica* was reduced by 57.51%. In a similar study, Ajaib et al. [86] assessed the anthelmintic activity of the extracts obtained from *D. ambrosioides* leaves collected in Pakistan against *Haemonchus contortus*. Chloroform extract took the minimum time for the paralysis and death of worms, with values of 12 and 17 h at 100 mg/mL concentration, whereas at 20 mg/mL, the maximum time taken for the paralysis and death of worms was also by chloroform extract, with values of 99 and 115 h. Zamilpa et al. [87] assessed the in vitro nematicidal effect of *D. ambrosioides n*-hexane cultivated in Mexico against *H. contortus* infective larvae. The results showed that the highest individual lethal in vitro effect (96.3%) was obtained with the *D. ambrosioides* extract at 72 h post-confrontation at 40 mg/mL, while the highest combined effect (98.7%) was obtained after 72 h at 40 mg/mL. The in vivo assay showed that the individual administration of the *D. ambrosioides* extracts reduced the parasitic burden in gerbils by 45.8%. Bernardes et al. [90] found that the essential oil obtained from leaves at a concentration of 20.0 µL/L was capable of killing 100% of adults of *Zabrotes subfasciatus* and demonstrated effective repellent activity at 0.8 µL/L air and Lethal Dose_50_. Niaz et al. [15] conducted a study to analyze anti-leishmanial activity against *Leishmania tropica* promastigotes using the essential oil obtained from fresh aerial parts through steam distillation of *D. ambrosioides* collected from Pakistan, which showed a high concentration of 4-carene and *o*-cymene. These authors reported that the essential oil of *D. ambrosioides* had a very potent anti-leishmanial activity with, an LC_50_ of Log_10_ 1.83 × 10^−6^ mg/mL. The low LC_50_ value indicates that the essential oil is very potent against *Leishmania tropica.* In a similar study, Pagotti et al. [24] explored the in vitro and/or in vivo trypanocidal (Chagas disease) activities of the essential oil obtained from *D. ambrosioides* collected from Brazil. *D. ambrosioides* oil was the most active against the trypomastigote and amastigote forms of *Trypanosoma cruzi* in vitro; the IC_50_ values were 8.7 and 12.2 µg/mL, respectively. The authors reported that this essential oil, which showed a high content of cis-piperitone oxide and *trans*-isoascaridole, had a high selectivity index (SI) for trypomastigote (SI = 33.2) and amastigote (SI = 11.7) forms. On day 7, in vivo treatment with *D. ambrosioides* at 20 mg/kg/day reduced parasitemia by 6.36%. Barros et al. [45] evaluated the essential oil of *D. ambrosioides*, extracted from the aerial parts of *D. ambrosioides* plants grown in Brazil, to determine its ability to inhibit the hatching of *Meloidogyne incognita* in vitro. They found that, at 0.5 mg/mL, the essential oil of *D. ambrosioides*, which showed a high content of α-terpinene and isoascaridole, induced mortality in the juvenile larvae of *Meloidogyne incognita* by more than 90%, with LC_50_ and LC_90_ values of 0.31 and 0.58 mg/mL, respectively. Soares et al. [91] reported that the essential oil (rich in cis-piperitone oxide and p-cymene) obtained from leaves of *D. ambrosioides* at concentrations of 25 and 12.5 µg/mL exhibited notable schistosomicidal action against *Schistosoma mansoni*. At this concentration, the essential oil killed 100% of adult worm pairs within 24 h. The LC_50_ values reported were 6.50, 3.66, and 3.65 µg/mL at 24, 48, and 72 h, respectively.

It is important to highlight that, when comparing the potential of *Dysphania ambrosioides* with other genera or species used for similar applications, it stands out for its anthelmintic effect, primarily due to the presence of ascaridole in its essential oil. Although other plants possess anthelmintic activity, *D. ambrosioides* is particularly noted for its use against root-knot nematodes. In addition, it has some pesticidal and insect-repellent properties (not very common in other genera) that are very valuable for organic farming. In addition, it grows easily in different conditions, thus enabling wide availability for processing at the industrial level. However, its toxicity is a significant drawback compared to other medicinal plants, especially essential oils; thus, its use requires caution and strong control of the doses applied to ensure the safe use of the treatment.

### 4.6. Other Biological Properties of Dysphania ambrosioides

Several studies have demonstrated the biological effects of *D. ambrosioides* essential oil and extracts. In reference to cancer, numerous studies reported that the extracts or components found in *D. ambrosioides* have a protective effect on the development and proliferation of some cancer cell lines. Therefore, Tauchen et al. [68] reported that *D. ambrosioides* extracts had an antiproliferative effect on a wide spectrum of cancer cells, including Caco-2, HT-29, and Hep-G2, with IC_50_ values of 29.2, 69.9, and 130.6 µg/mL, respectively. In another study, Shameem [46] analyzed the antiproliferative ability of *D. ambrosioides* essential oils against MCF-7 human mammary carcinoma cells and A549 human lung adenocarcinoma epithelial cells. They revealed that the highest antiproliferative activity was observed at 125 µg/mL in the A549 cell line, whereas the growth of the MCF-7 cell line was inhibited at 31.25 µg/mL. Pandiangan et al. [92] mentioned that the extracts obtained from the leaves of *D. ambrosioides* yielded an IC_50_ value of 0.105 µg/mL on P388 leukemia cells. Huang et al. [93] reported that the proliferation of human hepatocellular carcinoma cells SMMC-7721 was significantly inhibited by *D. ambrosioides* seed extracts, with IC_50_ values of 0.587 g/L, 0.360 g/L, and 0.361 g/L at 24 h, 36 h, and 48 h, respectively. Another biological activity of essential oils or extracts obtained from *D. ambrosioides* is their anti-inflammatory capacity. In this context, Rios et al. [94] mentioned that the hydroethanolic extract obtained from *D. ambrosioides* at a concentration of 5 mg/kg could inhibit nitric oxide and hydrogen peroxide production and, consequently, reduce inflammation. Ouadja et al. [62] revealed that both the essential oil and hydroethanolic extract obtained from the fresh leaves of *D. ambrosioides* significantly suppressed lipoxygenase (LOX) activity from 95.14 to 98.11 percent at the 1st, 5th, and10th min of the kinetics. Mokni et al. [43] reported that the essential oil obtained from the fresh leaves of *D. ambrosioides* had a remarkable antiviral activity against Coxsackie Virus-B4, with an IC_50_ value of 21.75 µg/mL.

### 4.7. Safety Margins of Dysphania ambrosioides Extracts and Essential Oils

While *D. ambrosioides* has a long history of traditional use, improper dosing or concentrated forms can be toxic. The distinction between traditional preparations (like infusions) and concentrated extracts or essential oils is vital.

In reference to the toxicity of *D. ambrosioides* crude extracts, Kandsi et al. [52] reported that the hydroethanolic extracts of *D. ambrosioides* flowers have an oral LD_50_ (lethal dose 50%, the dose at which 50% of the tested animals die) of 5 g/kg in acute toxicity studies in rats, while the aqueous and methanolic extracts obtained from leaves of *D. ambrosioides* yielded an LD_50_ greater than 2 g/kg also in rats. Similarly, Drioua et al. [95] revealed that the LD_50_ of crude extracts obtained from leaves of *D. ambrosioides* was greater than 2 mg/kg.

On the other hand, essential oil is generally considered more toxic than crude extracts due to its concentrated nature and higher content of active compounds, particularly ascaridole. A study carried out by Ez-Zriouli et al. [29] reported an LD_50_ of 0.5 mg/kg body weight for essential oil in rats, classifying it as a category four cytotoxic natural product at high doses. Adinci Kossi et al. [96] reported that an oral dose of D. ambrosioides essential oil at a concentration of 2 g/kg body weight induced death in rats within 24 h.

## 5. Conclusions and Perspectives

The use of artificial intelligence based on natural language processing (NLP), as applied in this study for literature review, represents an innovative tool to enhance the efficiency of scientific analyses. This approach warrants further exploration in future phytochemical and biomedical research. Using this methodology, it was possible to efficiently review and assess the relevance of 814 scientific published in just a few minutes, with a significant improvement over the time required for traditional search processes. Therefore, the usefulness of such a methodology is indisputable, marking a significant change in how state-of-the-art reviews are conducted across any scientific field. In this sense, future studies—regardless of their scientific field or purpose (e.g., projects, review papers, grant applications)—can leverage this tool to reduce selection biases and increase transparency. However, human oversight remains essential, especially in the final phase of the process, where the interpretation and in-depth analysis of selected information are critical. Additionally, this new tool is free to use and available to the entire scientific community, aligning with current open science principles adopted by many regulatory and funding entities, such as the Coalition for Advancing Research Assessment (CoARA) and the National Strategy for Open Science—Spain, which promote transparency in scientific research.

*Dysphania ambrosioides* has demonstrated significant ethnopharmacological potential due to its richness in bioactive compounds, particularly ascaridole, *p*-cymene, and α-terpinene. The essential oils and extracts of this plant exhibit strong antimicrobial activity against a variety of bacteria and fungi, including multidrug-resistant strains, highlighting its potential as a natural alternative to synthetic antibiotics. Additionally, its antioxidant properties, attributed to its high flavonoid and phenolic content, suggest potential applications against oxidative stress-related diseases. The plant also displays potent antiparasitic activity, particularly against nematodes and protozoa, supporting its traditional use in treating parasitic infections. Furthermore, its insecticidal and repellent effects position it as a promising candidate for eco-friendly pest control. Despite these promising findings, further research is needed to elucidate the precise mechanisms of action of its active compounds and to assess its safety and efficacy in clinical and agricultural applications. The standardization of extraction methods and toxicity studies are crucial for its integration into pharmaceutical and agricultural industries.

Future research on this topic should focus on the development and optimization of eco-friendly and efficient extraction methods, as well as the isolation and characterization of specific bioactive compounds. These compounds should then be used to elucidate precise mechanisms of action—even investigating underexplored therapeutic potentials—through advanced pharmacological studies. It is also crucial to conduct rigorous toxicological assessments to establish safe therapeutic windows and administration routes. Lastly, large-scale evaluations for specific applications should be performed. Successful execution of these research directions holds the potential not only to scientifically validate and optimize existing traditional uses but also to uncover entirely novel pharmaceutical applications. This could lead to the discovery of new drug candidates derived from natural sources, thus significantly contributing to addressing unmet medical needs and diversifying the global pharmacological arsenal.

## Figures and Tables

**Table 1:** Chemical composition of essential oils obtained from different parts of *Dysphania ambrosioides*.

Extracts	Part of the Plant	Compound	Concentration (%)	References
Essential oil	Leaves	Ascaridole-glycol Linalool acetate Dihydrocitronellol acetate	10.58 11.26 19.53	[22]
Essential oil	Leaves	α-Terpinene *p*-Cymene Carvacrol Ascaridole	30.50 17.30 16.20 15.10	[23]
Essential oil	Leaves	*Cis*-Piperitone oxide *Trans*-Isoascaridole *p*-Cymene	30.30 18.20 13.20	[24]
Essential oil	Leaves	β-Cymene 5-Isopropyl-6-methyl-hepta-3,5-dien-2-ol	47.10 19.20	[25]
Essential oil	Leaves and Stems	α-Terpinene *p*-Cymene *Trans*-Chrysanthenyl acetate	59.7 22.8 13.8	[26]
Essential oil	Leaves	Ascaridole *p*-Cymene	49.77 42.32	[27]
Essential oil	Leaves	Ascaridole α-Terpinene Ascaridole epoxide	15.13 54.09 9.77	[28]
Essential oil	Leaves	Ascaridole *p*-Cymene	35.50 47.20	[7]
Essential oil	Aerial Parts	α-Terpinene Ascaridole *p*-Cymene	53.4 17.7 12.1	[46]
Essential oil	Aerial Parts	*p*-Cymene 4-Carene α-Cyclogeraniol acetate	31.72 27.34 16.90	[30]
Essential oil	Aerial Parts	α-Terpinene *p*-Cymene	72.5 20.6	[31]
Essential oil	Aerial Parts	4-Carene α-Cyclogeraniol acetate *Trans*-β-Terpinyl butanoate	50.50 22.64 31.13	[32]
Essential oil	Leaves	δ-3-Carene *p*-Cymene	61.51 14.67	[33]
Essential oil	Aerial Parts	Ascaridole *Trans*-Ascaridole glycol *p*-Cymene	31.20 5.60 36.30	[34]
Essential oil	Aerial Parts	Isoascaridole α-Terpinene 2,3-Dehydro-1,4-cineole	15.30 15.20 55.00	[35]
Essential oil	Aerial Parts	Isoascaridole *Trans*-Ascaridole glycol *p*-Cymene	6.33 10.07 22.40	[36]
Essential oil	Aerial Parts	*o*-Cymene α-Terpinene Nona-3,5-dien-2-ol	39.20 36.80 10.00	[19]
Essential oil	Aerial Parts	*p*-Cymene α-Terpinene *cis*-Ascaridole	19.30 13.20 38.10	[37]
Essential oil	Aerial Parts	*(Z)*-Ascaridole *(E)*-Ascaridole *p*-Cymene	87 5.04 4.83	[38]
Essential oil	Aerial Parts	α-Terpinene Ascaridole *p*-Cymene	23.77 14.48 12.22	[39]
Essential oil	Aerial Parts	*p*-Cymene δ-3-Carene	14.70 61.50	[40]
Essential oil	Aerial Parts	Ascaridole Isoascaridole *p*-Cymene	16.30 51.00 6.70	[41]
Essential oil	Aerial Parts	*o*-Cymene (+)-4-Carene	41.46 56.59	[14]
Essential oil	Aerial Parts	Ascaridole *m*-Cymene	60.33 22.17	[42]
Essential oil	Whole Plant	*p*-Cymene α-Terpinene	49.60 26.81	[43]
Essential oil	Whole Plant	Ascaridole Isoascaridole *p*-Cymene	87.30 8.40 3.30	[44]
Essential oil	Aerial Parts	α-Terpinene Isoascaridole Ascaridole	37.17 20.48 14.83	[45]

**Table 2:** Antibacterial properties of essential oils and extracts obtained from different parts of *Dysphania ambrosioides*.

Extracts	Part of the Plant	Main Compound	Microorganism	MIC (µg/mL)	Ref.
Essential oil	Leaves	*o*-Cymene α-Terpinene	*Escherichia coli* *Staphylococcus aureus* *Pseudomonas aeruginosa* *Bacillus subtilis*	10 10 20 20	[5]
Essential oil	Aerial Parts	4-Carene *Trans*-β-Terpinyl butanoate	*E. coli* *S. aureus* *Enterococcus faecalis*	6 12 18	[32]
Essential oil	Leaves	β-Cymene	*S. aureus* *P. aeruginosa*	10 10	[25]
Essential oil	Aerial Parts	*o*-Cymene α-Terpinene	*E. coli* *B. subtilis*	7.8 3.9	[37]
Essential oil	Leaves	α-Terpinene Ascaridole	*E. coli* *S. aureus* *P. aeruginosa*	1024 256 512	[28]
Essential oil	Aerial Parts	α-Terpinene Ascaridole	*E. coli* *S. aureus* *P. aeruginosa* *Klebsiella pneumoniae*	310 1250 10,000 20,000	[40]
Essential oil	Aerial Parts	δ-3-Carene *p*-Cymene	*Microcystis aeruginosa*	3120	[41]
Essential oil	Aerial Parts	*p*-Cymene 4-Carene	*E. coli* *S. aureus* *P. aeruginosa*	90 120 120	[30]
Essential oil	Leaves	No Determined	*S. aureus*	1024	[55]
Essential oil	Leaves	α-Terpinene	*S. aureus*	1024	[56]
Essential oil	Aerial Parts	*cis*-Ascaridole *m*-Cymene	*P. aeruginosa* *Bacillus subtilis*	19 19	[43]
Ethanolic extract	Stem	Rutin Quercetin	*B. subtilis*	11.1	[12]
Ethanolic extract	Leaves	No determined	*E. coli* *S. aureus*	25,000 25,000	[57]
Ethanolic extract	Leaves	No determined	*Clostridioides difficile*	3900	[58]
Chloroform extract	Leaves	Quercetin Chrysin	*S. aureus* *Enterococcus faecalis*	4290 4290	[49]
Ethanolic extract	Aeria Parts	No determined	*E. coli* *E. faecalis*	1094 4375	[59]
Ethanolic and methanolic extracts	Aeria Parts	No determined	*E. coli* *S. aureus* *P. aeruginosa* *B. subtilis*	9 9 43 9	[60]

MIC: minimum inhibitory concentration.

**Table 3:** Antioxidant properties of essential oils and extracts obtained from different parts of *Dysphania ambrosioides*.

Extracts	Part of the Plant Used	Main Component	Methodology	Quantity	Reference
Ethyl Acetate extract	Aerial Part	No determined	FRAP ABTS	12.90 mg/mL 4.56 mg/mL	[66]
Aqueous extract	Leaves	16-methyl-heptadecane-1,2-diol Phytol	FIC FRAP DPPH	IC_50_ 20.98 mg/mL 64.19 mg/AAE g IC_50_ 1.39 mg/mL	[11]
Methanolic extract	Seeds	Rutin	DPPH ABTS FRAP	IC_50_ 110.7 µg/mL 110.6 µg AAE/mg 94.30 µg AAE/mg	[63]
Aqueous extract	Fruits	No determined	ABTS FIC	8.25 mM TE/g 78% quelation	[60]
*n*-Butanol ethyl acetate extracts	Leaves	Caffeic acid Coumarin Kaempferol	DPPH	IC_50_ 2.98 mg/mL IC_50_ 16.48 mg/mL	[54]
Methanolic extract	Leaves	Rutin Quercetin	DPPH	IC_50_ 130.7 µg/mL	[12]
Methanolic extract	Leaves	No determined	FRAP ABTS	0.141 µM TE/g 0.224 mg AAE/g	[67]
Hydroethanolic extract	Flowers	Syringic acid Quercetin Kaempferol	DPPH β-Carotene/linoleic acid FRAP	IC_50_ 166.47 µg/mL IC_50_ 57.04 µg/mL IC_50_ 231.05 µg/mL	[1]
Aqueous extract	Aerial Part	No determined	DPPH ORAC	IC_50_ 80.6 µg TE /mL IC_50_ 687.3 µg TE/mL	[68]
Essential oil	Aerial Part	α-Terpinene Ascaridole	DPPH	30.182 mg TE/g oil	[29]
Essential oil	Leaves	No determined	DPPH FRAP ABTS	1.59 mg AAE/g 8.36 mg AAE/g 2.11 mg AAE/g	[59]
Essential oil	Stems	4-Carene α-Cyclogeraniol Acetate	FRAP ABTS	IC_50_ 309.45 µg/mL IC_50_ 147.99 µg/mL	[32]
	Flower	*trans*-β-TerpinylButanoate 4-Carene	DPPH β-Carotene/linoleic acid	IC_50_ 158.15 µg/mL IC_50_ 266.25 µg/mL	
Essential oil	Leaves	α-Terpinene Ascaridole	DPPH	IC_50_ 1024 µg/mL	[28]
Essential oil	Aerial Part	α-Terpinene Ascaridole	DPPH β-Carotene/linoleic acid FRAP	IC_50_ 4.00 mg/mL IC_50_ 3.03 µg/mL IC_50_ 6.02 µg/mL	[40]
Essential oil	Leaves	α-Terpinene α-Terpinenyl Acetate	DPPH	IC_50_ 1.74 mg/mL	[69]

FRAP: ferric reducing power assay; ABTS: (2,2′-azino-bis(3-ethylbenzothiazoline-6-sulphonic acid)) method; DPPH: 2,2-diphenyl-1-picrylhydrazyl assay; FIC: ferrous ion chelating assay; TE: Trolox equivalent. IC_50_: its ability to eliminate 50% of free radicals; AAE: ascorbic acid equivalents.

**Table 4:** Insecticidal and repellent activities of essential oils and extracts obtained from different parts of *Dysphania ambrosioides*.

Extracts	Part of the Plant Used	Main Compounds	Quantity	Insects	Reference
Essential oil	Leaves	No determined	1 µL/L air	*Periplaneta americana*	[73]
Essential oil	Aerial Parts	Terpinolene *p*-Cymene	1.02 µL/L air	*Dactylopius opuntiae*	[74]
Essential oil	Aerial Parts	*cis*-Ascaridole *p*-Cymene	3.125 µL/L	*Culex quinquefasciatus*	[38]
Essential oil	Aerial Parts	*p*-Cymene Ascaridole	62.5 mg/L 10 mg/L	*Aedes aegypti* *Anopheles gambiae*	[34]
Essential oil	Aerial Parts	*o*-Cymene α-Terpinene	0.75 mg/mL	*Culex pipiens*	[37]
Essential oil	Whole Plant	No determined	66.81 mg/L	*Plutella xylostella*	[44]
Essential oil	Leaves	δ-3-Carene *p*-Cymene	0.04 µL/cm^2^	*Tribolium confusum*	[33]
	Inflorescences	δ-3-Carene *p*-Cymene	0.05 µL/cm^2^		
Essential oil	Leaves	No determined	0.50 mg/m^2^	*Callosobruchus maculatus*	[75]
Essential oil	Fresh Leaves	Ascaridole *p*-Cymene	LC_50_ 17.74 µg/cm^2^	*Alphitobius diaperinus*	[76]
Extracts	Aerial Parts	No determined	50 g/L	*Dactylopius opuntiae*	[77]
Extracts	Aerial Parts	No determined	10 g/L	*Bemisia tabaci*	[78]
Extracts	Aerial Parts	No determined	200 g/L	*Spodoptera frugiperda*	[79]
Extract	Stem and Leaves	No determined	500 mg/mL	*Scyphophorus scupunctatus*	[80]

LC_50_: lethal concentration 50%.

**Table 5:** Antiparasitic activities of essential oils and extracts obtained from different parts of *Dysphania ambrosioides*.

Extracts	Part of the Plant	Main Compound	Concentration	Parasites	Reference
Essential oil	Fruit and Seeds	*(Z)*-Ascaridole *E*-Ascaridole	307 µg/mL	*Meloidogyne incognita*	[45]
Essential oil	Aerial Part	4-Carene *o*-Cymene	4.74 mg/mL	*Leishmania tropica*	[15]
Essential oil	Aerial Part	*cis*-Piperitone oxide *trans*-Isoascaridole	8.7 µg/mL	*Trypanosoma cruzi*	[24]
Essential oil	Aerial Part	No determined	50 µL/mL	*Ancylostoma* spp.	[39]
Essential oil	Aerial Part	*p*-Cymene α-terpinene	0.037 µL/g	*Rhipicephalus lunulatus*	[85]
Essential oil	Flower	Isoascaridole Ascaridole	0.041 µL/mL	*Meloidogyne chitwoodi*	[42]
Extracts	Aerial Part	No determined	20 mg/mL	*Haemonchus contortus*	[86]
Extracts	Aerial Part	No determined	0.6 mg/mL	*Haemonchus contortus*	[87]
Extracts	Aerial Part	No determined	400 mg/mL	*Rhipicephalus microplus*	[88]
Extracts	Leaves	No determined	300 mg/mL	*Meloidogyne javanica*	[82]
Extracts	Leaves	No determined	50 mg/mL	*Meloidogyne enterolobi*	[89]
Extracts	Whole Plant	Rutin Quercetin	1 mg/mL	*Leishmania tropica*	[12]

## Data Availability

Data are contained within the article and Supplementary Materials.

## Supplementary Materials

The following supporting information can be downloaded at: https://www.mdpi.com/article/10.3390/plants14131903/s1, Figure S1: Quantitative distribution of the papers in each of the 19 clusters (numbered from 0 to 18); Figure S2. Word clouds of the 75 most common words in each cluster (the number of the cluster is indicated at the top of each word cloud); Figure S3. Frequency bar chart of the 15 most common (the highest frequency appearance) words in each of the 19 clusters (numbered from 0 to 18). Figure bars for the 15 top words (the highest frequency appearance) for each cluster (19 clusters numbered from 0 to 18 and identified at the top of each figure); Figure S4. Scatter plot of the principal component analysis results for the 814 papers included in the datasheet of the initial bibliographic research. Each cluster is represented by a different color, corresponding the black points to the centroids of each cluster; Figure S5. Distribution of the 19 clusters over time (2000–2024). Table S1: Data, IA-based tools, and results obtained during the review of “Epazote (*Dysphania ambrosioides*): A phytochemical treasure with multiple applications: A review”.
