# Supplementary material for: Essential Oils and Extracts from Epazote (Dysphania ambrosioides): A Phytochemical Treasure with Multiple Applications
Source: Plants (Basel). 2025 Jun 20;14(13):1903. doi: 10.3390/plants14131903 (PMC12251798; doi:10.3390/plants14131903)
Supplement: Supplementary file 1 [file plants-14-01903-s001.zip › Figure S2.pdf]

Cluster 0

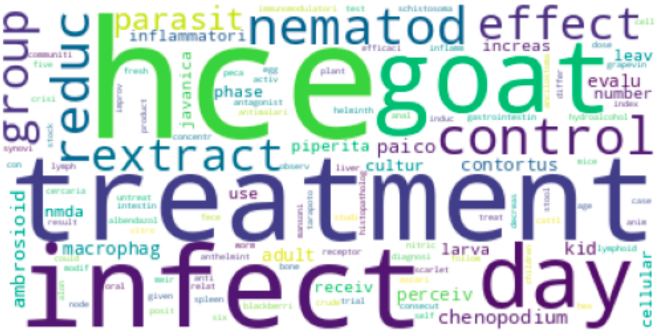

Cluster 1

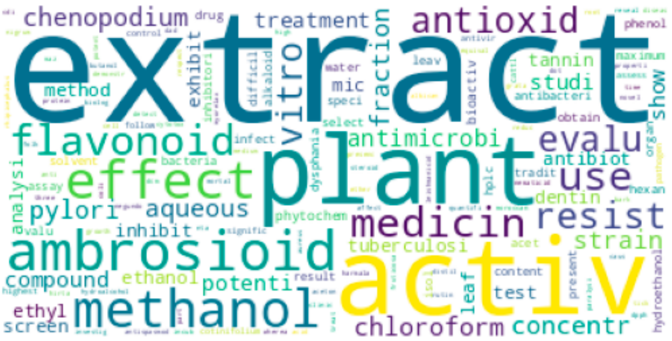

Cluster 2

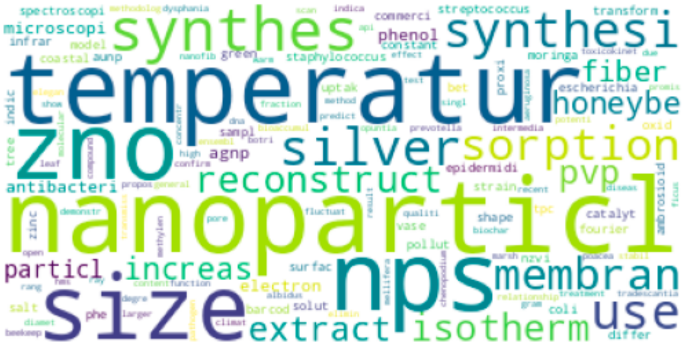

Cluster 3

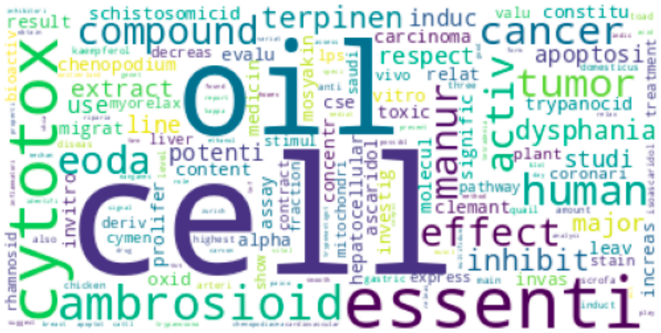

Cluster 4

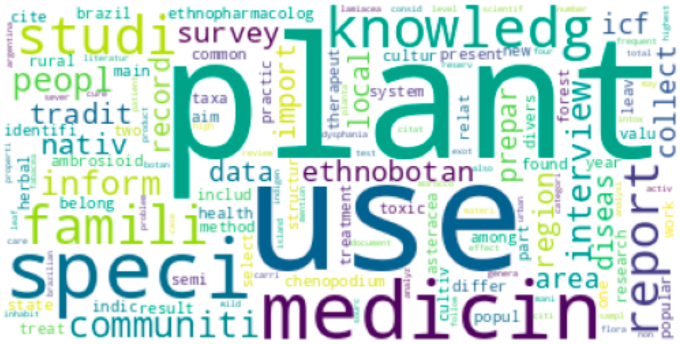

Cluster 5

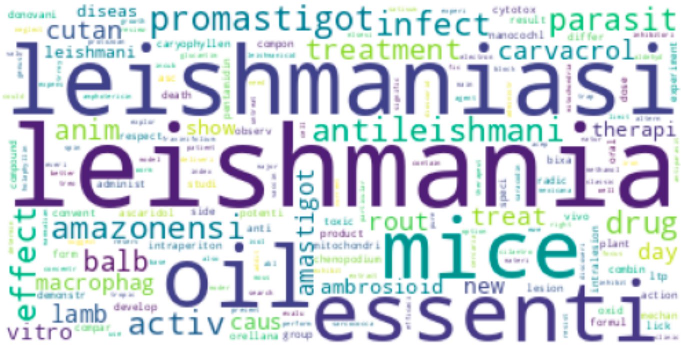

Cluster 6

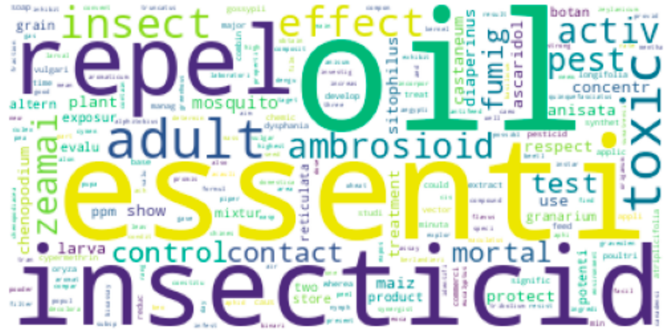

Cluster 7

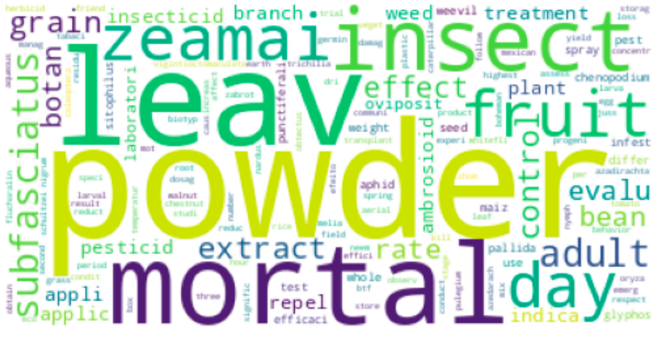

Cluster 8

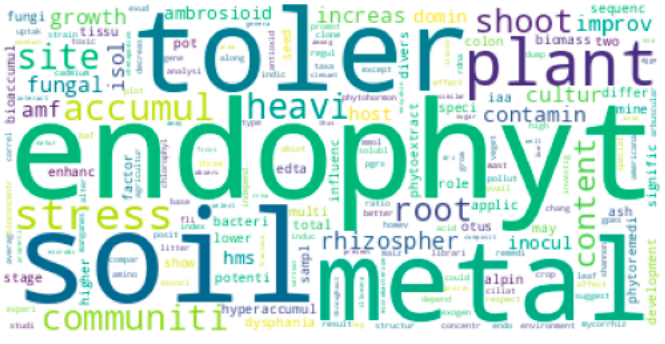

Cluster 9

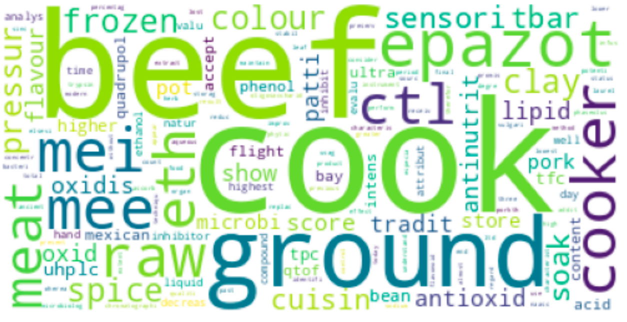

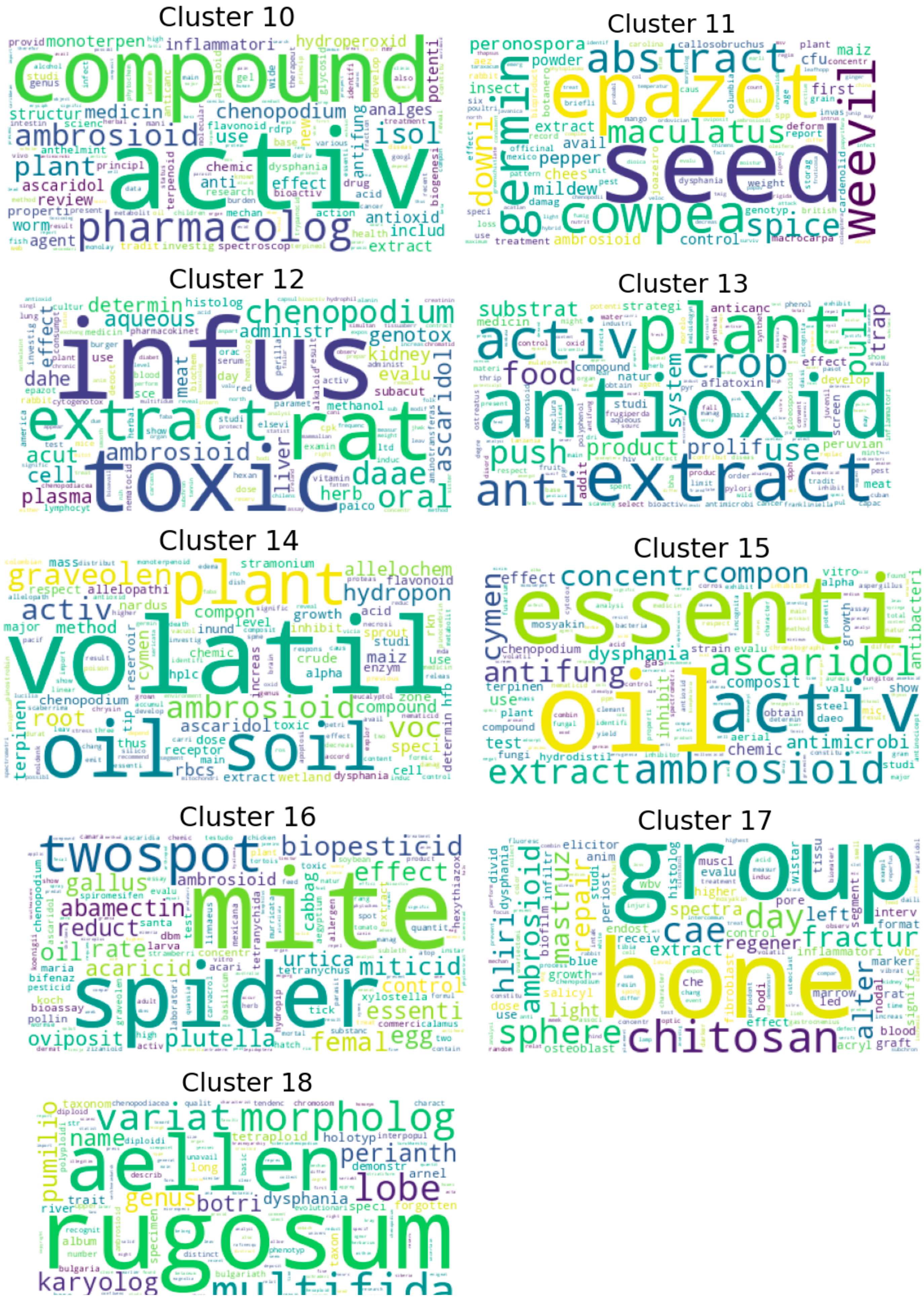

Figure S2. Word clouds of the 75 most common words in each cluster (then number of the cluster is indicated at the top of each word cloud)
