# Supplementary material for: Essential Oils and Extracts from Epazote (Dysphania ambrosioides): A Phytochemical Treasure with Multiple Applications
Source: Plants (Basel). 2025 Jun 20;14(13):1903. doi: 10.3390/plants14131903 (PMC12251798; doi:10.3390/plants14131903)
Supplement: Supplementary file 1 [file plants-14-01903-s001.zip › Figure S3.pdf]

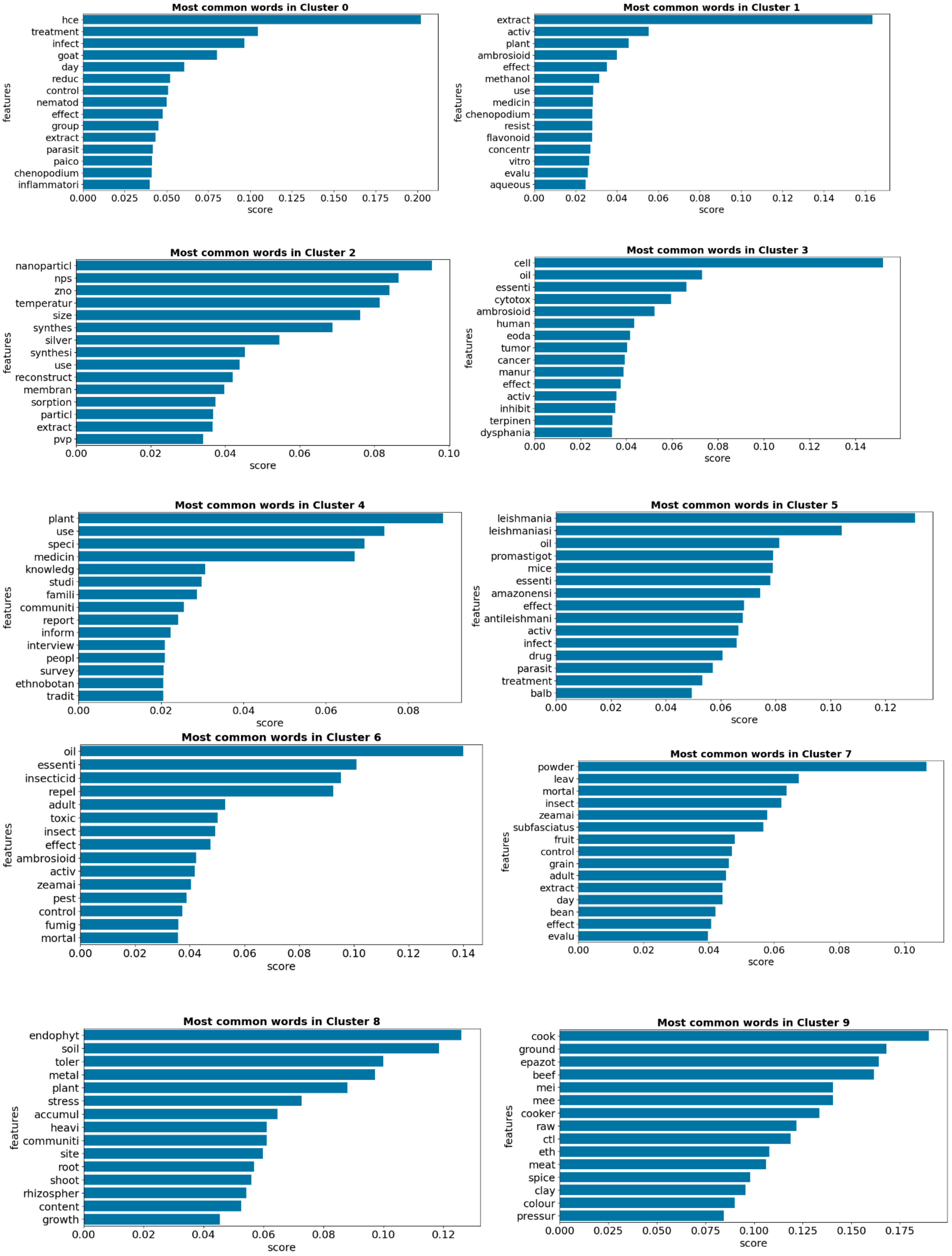
Most common words in Cluster 0
hce
treatment
infect
goat
day
reduc
control
nematod
effect
group
extract
parasit
paico
chenopodium
inflammatori
features
score
0.000 0.025 0.050 0.075 0.100 0.125 0.150 0.175 0.200
Most common words in Cluster 1
extract
activ
plant
ambrosioid
effect
methanol
use
medicin
chenopodium
resist
flavonoid
concentr
vitro
evalu
aqueous
features
score
0.00 0.02 0.04 0.06 0.08 0.10 0.12 0.14 0.16
Most common words in Cluster 2
nanoparticl
nps
zno
temperatur
size
synthes
silver
synthesi
use
reconstruct
membran
sorption
particl
extract
pvp
features
score
0.00 0.02 0.04 0.06 0.08 0.10
Most common words in Cluster 3
cell
oil
essenti
cytotox
ambrosioid
human
eoda
tumor
cancer
manur
effect
activ
inhibit
terpinen
dysphania
features
score
0.00 0.02 0.04 0.06 0.08 0.10 0.12 0.14
Most common words in Cluster 4
plant
use
speci
medicin
knowledg
studi
famili
communiti
report
inform
interview
peopl
survey
ethnobotan
tradit
features
score
0.00 0.02 0.04 0.06 0.08
Most common words in Cluster 5
leishmania
leishmaniasi
oil
promastigot
mice
essenti
amazonensi
effect
antileishmani
activ
infect
drug
parasit
treatment
balb
features
score
0.00 0.02 0.04 0.06 0.08 0.10 0.12
Most common words in Cluster 6
oil
essenti
insecticid
repel
adult
toxic
insect
effect
ambrosioid
activ
zeamai
pest
control
fumig
mortal
features
score
0.00 0.02 0.04 0.06 0.08 0.10 0.12 0.14
Most common words in Cluster 7
powder
leav
mortal
insect
zeamai
subfasciatus
fruit
control
grain
adult
extract
day
bean
effect
evalu
features
score
0.00 0.02 0.04 0.06 0.08 0.10
Most common words in Cluster 8
endophyt
soil
toler
metal
plant
stress
accumul
heavi
communiti
site
root
shoot
rhizospher
content
growth
features
score
0.00 0.02 0.04 0.06 0.08 0.10 0.12
Most common words in Cluster 9
cook
ground
epazot
beef
mei
mee
cooker
raw
ctl
eth
meat
spice
clay
colour
pressur
features
score
0.000 0.025 0.050 0.075 0.100 0.125 0.150 0.175

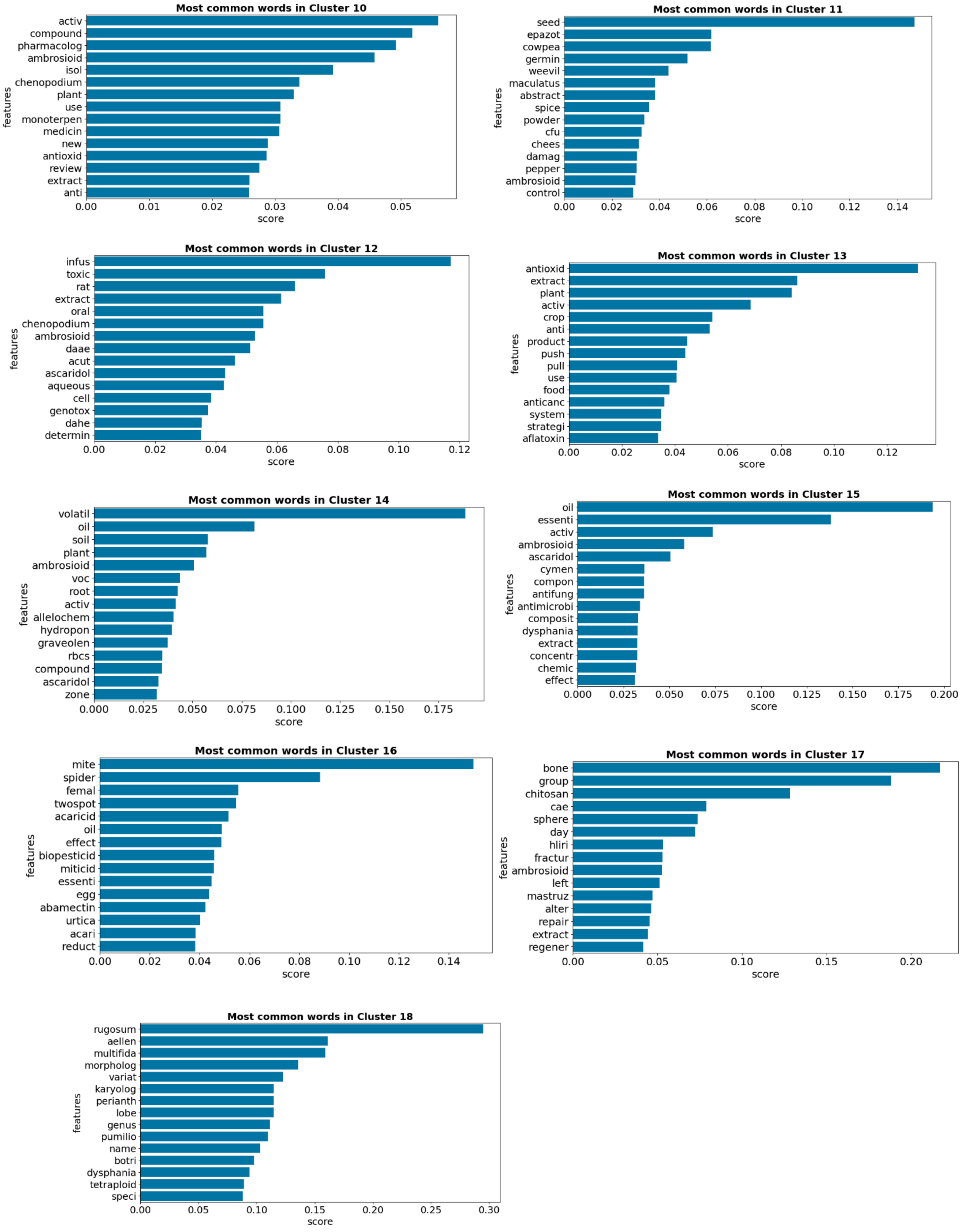

Figure S3. Frequency bar chart of the 15 most common (the highest frequency appearance) words in each of the 19 clusters (numbered from 0 to 18). Figure bars for the 15 top words (the highest frequency appearance) for each cluster (19 clusters numbered from 0 to 18 and identified at the top of each figure).
