# Supplementary material for: Essential Oils and Extracts from Epazote (Dysphania ambrosioides): A Phytochemical Treasure with Multiple Applications
Source: Plants (Basel). 2025 Jun 20;14(13):1903. doi: 10.3390/plants14131903 (PMC12251798; doi:10.3390/plants14131903)
Supplement: Supplementary file 1 [file plants-14-01903-s001.zip › Figure S4.pdf]

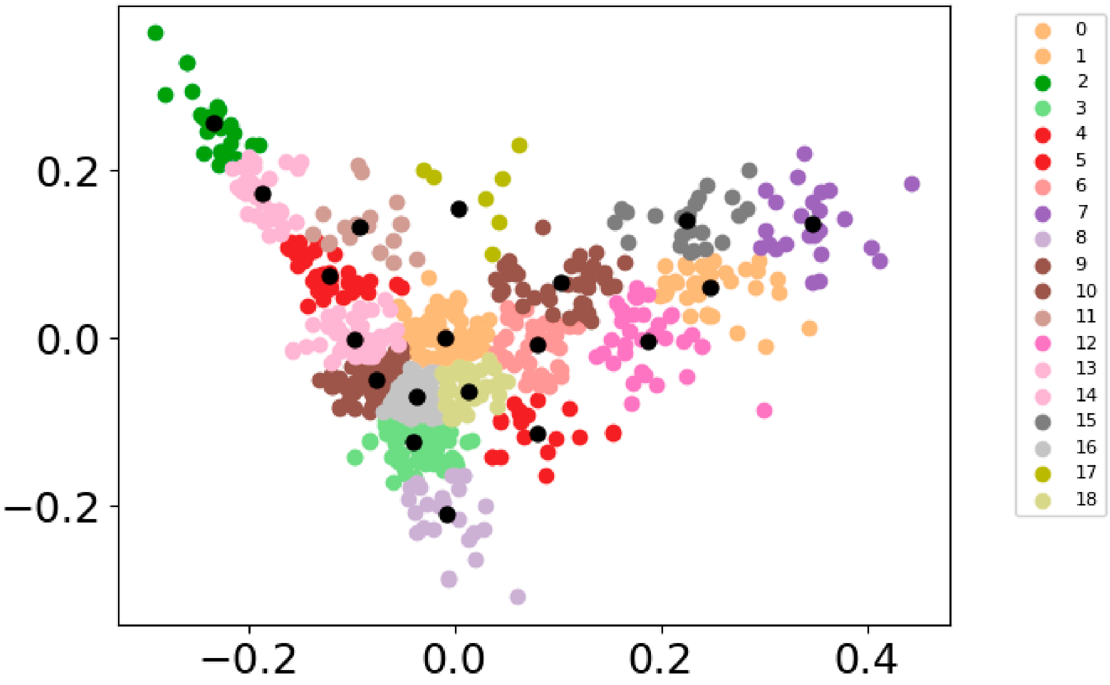

Figure S4. Scatter plot of the Principal Component Analysis results for the 814 papers included in the datasheet of the initial bibliographic research. Each cluster is represented by a different color; corresponding the black points to the centroids of each cluster.
