# Supplementary material for: Essential Oils and Extracts from Epazote (Dysphania ambrosioides): A Phytochemical Treasure with Multiple Applications
Source: Plants (Basel). 2025 Jun 20;14(13):1903. doi: 10.3390/plants14131903 (PMC12251798; doi:10.3390/plants14131903)
Supplement: Supplementary file 1 [file plants-14-01903-s001.zip › Table S1.pdf]

Table S1.- Data, IA-based tools and results obtained during the review of Epazaote (*Dysphania ambrosioides*): A phytochemical treasure with multiple applications. A review

Action	Link to data, IA-based tools and results
Scopus file	
First analysis of the initial bibliographic search	https://colab.research.google.com/drive/15vydY5OhrOa-3_gJCc51LyVGbEaO9MXv#scrollTo=Tw7-MF4RL27p
Second round: Analysis of Subcluster 0, 1, 3, 6, 7, 10, and 15	https://colab.research.google.com/drive/1TEEe8-tLkJ6mCsSjFzLuzlyyvQvHD28R
