# Supplementary figures and images for: Essential Oils and Extracts from Epazote (Dysphania ambrosioides): A Phytochemical Treasure with Multiple Applications
Source: Plants (Basel). 2025 Jun 20;14(13):1903. doi: 10.3390/plants14131903 (PMC12251798; doi:10.3390/plants14131903)

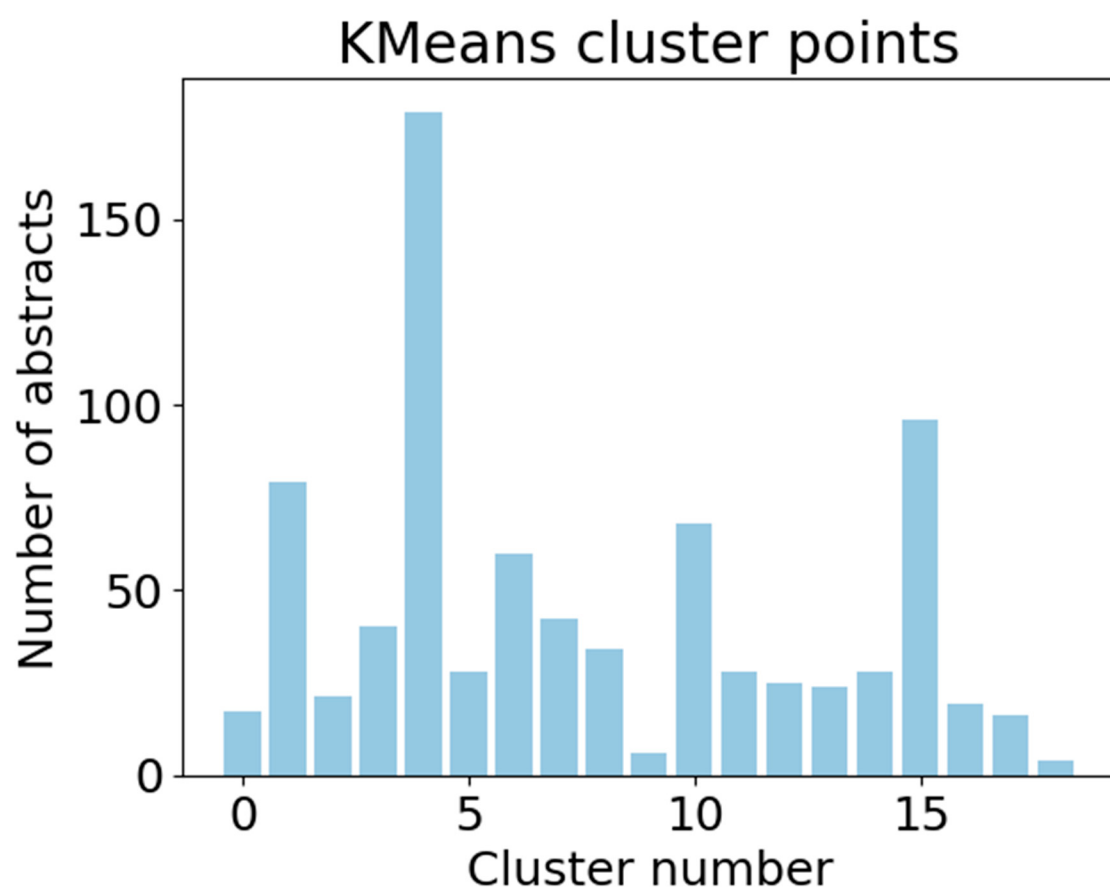

Figure S1. Quantitative distribution of the papers in each of the 19 clusters (numbered from 0 to 18).

Supplement: Supplementary file 1 [file plants-14-01903-s001.zip › Figure S1.pdf]

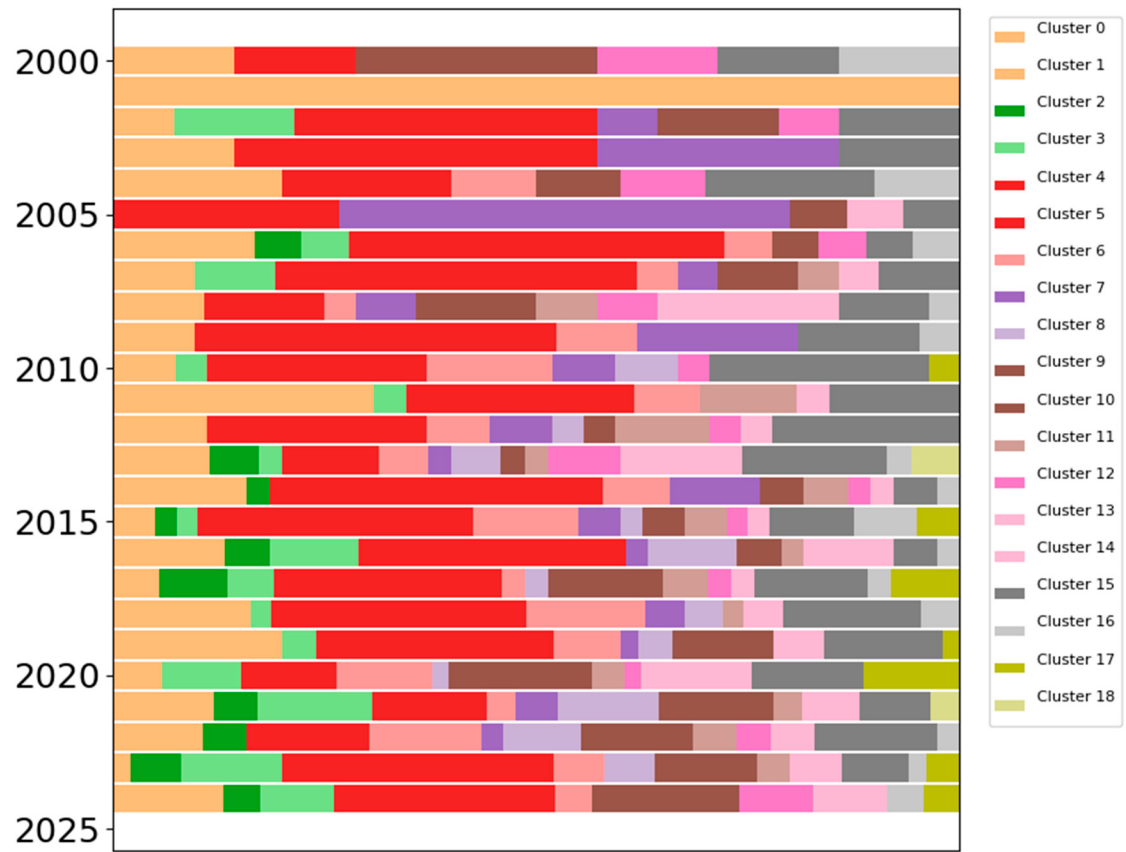

Figure S5. Distribution of the 19 clusters over the time (2000–2024).

Supplement: Supplementary file 1 [file plants-14-01903-s001.zip › Figure S5.pdf]
